# Neuropsychological rehabilitation program and behavioral disturbances in early-stage Alzheimer patients

**DOI:** 10.1590/S1980-57642009DN20200012

**Published:** 2008

**Authors:** Vera Lúcia Duarte Vieira, Sônia Maria Dozzi Brucki, Anna Luiza Costa Marques Martins, Fabíola Canali, Luciano Gois Vasconcelos, Maira Okada de Oliveira, Beatriz Schlecht Bittencourt, Lúcia Julieta Tonato Leite, Jacqueline Abrisqueta-Gomez, Orlando Francisco Amodeo Bueno

**Affiliations:** 1 Post graduante students, psychologist of Department of Psychobiology of federal University of São Paulo,Brazil.; 2 Medical Doctor of Department of Psychobiology of federal University of São Paulo,Brazil.; 3 Collaborator of Department of Psychobiology of federal University of São Paulo,Brazil.; 4 Adjunct Professor and Head of Department of Psychobiology of federal University of São Paulo,Brazil.

**Keywords:** dementia, caregivers, behavioral disorders, non-pharmacological interventions

## Abstract

Alzheimer’s disease (AD) is the most frequent cause of dementia and cholinesterase inhibitors are the available treatment in the mild stage. However cognitive rehabilitation has shown satisfactory results when combined with pharmacological treatment. Behavioral alteration is common in AD patients, which burdens caregivers and raises the risk of institutionalization. Providing caregivers guidance may enable them to assure better quality of life for patient and caregiver and lower institutionalization rates.

**Objective:**

To evaluate the effects of a neuropsychological rehabilitation program (NRP) combined with pharmacological treatment in early stage AD patients.

**Methods:**

We studied 12 AD patients (6 women), average age 75.42 (6.22) with 9.58 (5.6) years education in use of stable doses of cholinesterase inhibitors. Cognitive performance was evaluated using Mini-Mental State Examination (MMSE) and Alzheimer´s Disease Assessment Scale-cognitive (ADAS-Cog). Caregivers responded to Neuropsychiatric Inventory (NPI) and Functional Activities Questionnaire (FAQ) at initial evaluation (T1), and after 8 months of rehabilitation program (T2). The program comprised two sessions every week and family guidance every fortnight.

**Results:**

MMSE (T1:23.25 (1.82)/T2:23.42 (2.81); ADAS-Cog (T1:17.11 (6.73)/T2:21.2 (8.59); NPI (T1:23.42 (23.38)/T2:19.83 (17.73); FAQ (T1:10.67 (7.24)/T2: 13.92 (6.92).

**Conclusions:**

These results show the importance of providing guidance and support for caretakers. Study limitations were the small number of patients and absence of a control group with only drug treatment to compare with combined pharmacological and rehabilitation treatments.

## Abstract (pt)

A doença de Alzheimer é a demência mais freqüente, o tratamento disponível específico, no estágio leve é com droga anticolinesterásico. Por outro lado a reabilitação cognitiva tem apresentado resultados satisfatórios quando associado ao tratamento farmacológico. É comum alteração de comportamento em pacientes com DA, o que contribui para uma maior sobrecarga do cuidador aumentando o risco de institucionalização. Cuidadores orientados sobre a doença pode permitir uma melhor qualidade de vida para ambos diminuindo a taxa de institucionalização.

**Objetivos:**

Avaliar os efeitos de um programa de reabilitação neuropsicológica (PRN) associado ao tratamento farmacológico de pacientes em fase leve da DA.

**Métodos:**

12 pacientes com DA (6 mulheres) com idade média de 75.42 (6.22) e 9.58 (5.6) anos de escolaridade e dose estável de anticolinesterásicos foram avaliados com o Mini-Exame do Estado Mental (MEEM) e Alzheimer's Disease Assessment Scale-cognitive (ADAS-Cog) e os cuidadores responderam NPI e FAQ na avaliação inicial (T1) e depois de 8 meses do PRN (T2). O PRN foi composto por duas sessões semanais e Orientação Familiar a cada 15 dias.

**Conclusões:**

Estes resultados nos mostram a importância do trabalho desenvolvido com cuidadores, propiciando orientação e suporte. Limitações aos resultados podem estar presentes pelo número reduzido de pacientes, ausência de um grupo controle que permitisse a comparação do tratamento farmacológico isolado com tratamento farmacológico e reabilitação em conjunto.

The numbers of families affected by dementia has risen drastically in recent decades. Care-giver guidance may lead to improved quality of life for both and less patient time in hospital, mainly by reducing behavioral disturbances. The publication “Guidelines for managing Alzheimer’s Disease”,^1^ suggests that a treatment plan should be developed when the diagnosis has been made. This plan should include: pharmacological therapy (with cholinesterase inhibitor to temporarily improve cognition or slow the rate of cognitive decline), managing possible co-morbidity, treatment of behavioral symptoms and mood disorders and finally support for patients and their relatives.

In addition, following recommendations and suggestions of the Cognitive Neurology and Aging Department of the Brazilian Academy of Neurology,^2^ cognitive rehabilitation may be indicated as supplementary treatment (combined with medicamentous therapy) in patients with mild to moderate DA since two studies published in Brazil^3-4^ have demonstrated its positive effects.

Had previously noted that neuropsychological rehabilitation should not only work for cognitive improvement but also seek to enable patient and family to deal with everyday life, and work around, reduce or overcome cognitive, emotional and social impairments to obtain significant improvement in quality of life.^5^

Behavioral disturbances are very frequent in AD and may take place from the initial phases. Their frequency varies across studies, but in the initial phases the most frequently reported symptoms are: apathy (70%), anxiety^6^ (48%), depression (50%) and irritability (42%).^7^ According to psychotic behavior is more frequent in the moderate to severe stages.^6^ These disorders are associated with higher probability of institutionalization and increased numbers of medications used by these patients. Another fundamental aspect is the subjective burden for caregivers when patients have behavioral disorders.

## Objectives

Verify the effect of a family guidance program by administering questionnaires containing and functional and behavioral scales to caregivers of mild AD patients. Note that the measure of success of a rehabilitation strategy is evaluated in daily life rather than in the therapeutic environment since the aim is to have patients become more adapted and functional in their own surroundings.

## Methods

### Patients

Subjects were 12 patients (six women) average age 75.42 (6.22) and 9.58 (5.60) years schooling diagnosed as probable AD using the NINCDS-ADRDA criteria^4^ and AD diagnosis using DSM-IV criteria 5. Subjects were recruited at the UNIFESP/ EPM hospital complex (Hospital São Paulo and Hospital Santa Marcelina). Patients were first subjected to medical testing to confirm clinical diagnosis and indicate the stage of the disease, and to detect possible co-morbidities that might affect the research. Another inclusion criterion was having a family-member caregiver. The composition of the latter group in terms of family relations was as follows: spouses n=7, children n=3, sister n=1 and daughter-in-law n=1.

Family-member caregivers were informed of the research goal and procedure and provided signed consent for patient’s participation in the study, promised to take part in the orientation program, and were told that after three consecutive absences the patient and family-member caregiver would be removed from the study.

All underwent neurological and neuropsychological evaluation before and after eight months intervention and were taking stable doses of cholinesterase inhibitors.The following steps were taken to control the variations common to clinical studies:

(a) tests were administered in semi-random order, in other words, the neuropsychological evaluation procedure was divided into four blocks, maintaining a fixed order of tests within each block. Only the first block contained tracking tests and batteries. The other three blocks were administered in random order;
(b) test instructions were standardized and read by the examiner;
c) evaluation conditions were virtually to the same for all subjects (physical space, lighting, testing materials, and others);
d) examiners were trained to administer the tests prior to administering them.

### Cognitive and functional evaluation

Tests were conducted before and after a family guidance program:

- Mini-Mental State Examination^8-9^ – maximum score 30 points;
- ADAS-Cog ^10-11^– maximum 70 points;
- Bayer Daily Life Activity Scale (B-AVD)^12^ - consisting of 25 questions to evaluate the degree of patient difficulty when performing tasks, measured on a scale from 1 (no difficulty) to 10 (cannot do);
- Functional Activities Questionnaire (FAQ):^13^ questionnaire that evaluates functional activities with maximum score 30 points.

### Behavioral evaluation

- Neuropsychiatric Inventory^14^ which evaluates behavioral and psychological disturbances, such as delusions, hallucinations, agitation, dysphoria, anxiety, apathy, irritability, euphoria, disinhibition, aberrant motor behavior, night-time behavior disturbances, and delirious ideas (measures frequency and severity of each disturbance);
- The Revised Memory and Behavior Problems Checklist -^15^ this scale appraises the frequency of patient behavior problems and the reaction they provoke in caregivers The higher the score, the more frequent the behavioral problems and the worse the caregiver’s reaction to these problems.

Functional and behavioral evaluations were conducted using scales that caregivers responded to in groups. Presentation was through a projector and the study was conducted with advice from three professionals (two neuropsychologists and an occupational therapist), for two hours duration.

### Family guidance program (FG)

The eight-month program involved a fortnightly session lasting an hour and a half. The study was developed at the São Paulo Neuropsycology Center, Universidade Federal de São Paulo (UNIFESP).

FG consisted of:

- Information about AD in the form of a talk, referring mainly to cognitive and behavioral evolution and disturbances. It also covered available medication treatment;
- Guidance for relatives on how to approach and cope with cognitive and behavioral disturbances;
- Restructuring routines with relatives and implementing functional strategies. External supports were used (calendar, pages with personal data and diary), plus advice on how to improve communication with patients;
- Dietary guidelines;
- Family therapist guidance at the end of the period, comprising two sessions dealing with emotional issues raised by family caregivers in an attempt to join the interests of patients and caregivers.

### Statistical analysis

A significance level of 5% (0.050) was adopted for use of statistical tests in this study. The program used in analyses was the Statistical Package for Social Science” (SPSS).

Descriptive analyses were made for continuous category data (average, standard deviation, minimum and maximum). Pre- and post-treatment scores were compared using the Wilcoxon test.

The project was approved by the Research Ethics Committee, Universidade Federal de São Paulo. Informed consent forms were signed by the main caregivers and/or legal guardians.

## Results

Table 1 shows demographics. On comparing the first and last evaluations, we noted a relative stabilization of results (after 8 months of intervention) as shown in Table 2.

**Table 1:** Demographic characteristics.

	Mean	Standard deviation	Minimum value	Maximum value
Gender (n)	F (6) / M (6)	-	-	-
Age	75.42	75.42	64	84
Educational level	9.58	5.60	4	15

F: female; M: male.

**Table 2:** Comparisons between first and second scores on cognitive, behavior, and functional evaluations.

	Mean (first evaluation)	SD	Mean (second evaluation)	SD	Z	p value
MEEM	23.25	1.82	23.42	2.81	-0.197	0.844
ADAS-Cog	17.11	6.73	21.20	8.59	-1.255	0.209
Pfeffer	10.67	7.24	13.92	6.92	-1.735	0.083
NPI	23.42	23.38	19.83	17.73	-1.138	0.255
Bayer	101	45.27	107	53.33	-0.628	0.530

Wilcoxon test.

## Discussion

Our research findings corroborate those in the literature on behavior alteration in early-stage AD patients and suggest that non-pharmacological treatment may delay onset of behavioral and mood disturbances and enhance quality of life for these patients and their caregivers and consequently reduce institutionalization^16-18^. However it is important to note that patients in this study were submitted to individual and group intervention (once a week for each type) which may explain the stable cognitive, functional and behavioral scores from first to second evaluation. However note that The Revised Memory and Behavior Problems Checklist,^15^ which evaluates caregiver response to memory, behavior and humor alterations, also found an improvement in caregiver response to alterations presented by patients (Memory- Z= –0.314 And P=0.753; Behavior Z= –1.309 and p=0.474; Mood Z= –1.309 and p=0.191). These findings are compatible with those of Abrisqueta-Gomez et al.^3^

Note that behavioral alterations are directly related to worsening of patient functionality.^19,20^ Another extremely important point is the patient environment, which calls for a restructuring routine with implementation of functional strategies able to make patients more functional and better adapted patient to this environment. However we find this often meets with initial resistance from patients still in the early stage whose critical faculties and judgment are relatively intact. Therefore our guidance sessions included explanation and training on this point since caregivers also would their routines restructured. On this issue, we conducted a caregiver evaluation four months into the study, but the findings have yet to be analyzed.

Some studies also point that behavior alteration in patients may be associated with caregiver stress, depression and anxiety.^21-23^. On analyzing findings using The Revised Memory and Behavior Problems Checklist^15^, which evaluates caregiver responses to memory, behavior and mood alterations, we found improved caregiver response to alterations presented by patients (Memory- Z= –0.314 and p=0.753; Behavior Z= –1.309 and p=0.474; Mood Z= –1.309 and p=0.191). This finding matches those of Abrisqueta-Gomez et al.^3^ An important point is that patients present less behavioral alteration in the early stages.

After systematically reviewing studies on information and support for dementia patients’ caregivers, concluded that this was a significantly positive effect in relation to caregiver depression.^24^ Previous studies conducted at SARI showed the importance of evaluating these aspects before starting a rehabilitation program.^3^ Published a study of family caregivers in which A-B-C behavior modification technique was used (A–activator, B–behavior observed and C–consequence).^21-25^ In this study, the caregiver was taught to identify alteration triggering situations and then trained to monitor patient’s daily behavior and make notes when the target behavior occurred, identifying situations or whatever was associated with this occurrence. Caregivers were then taught behavioral change strategy. In our own study this technique was not used in full but our guidance sessions for caregivers did include behaviors that might elicit alterations in patients. We also talked about how to improve communication, another factor that may lead to patient behavior alterations since there is often decline in language, mostly in naming.^26^ Guidance included behavioral tips for caregivers such as “always look at the patient when talking to them, use short and simple sentences.”

In light of our findings, we may conclude that there is evidence of the importance of work developed with caregivers and or family to ensure guidance and support: being informed of the course of the disease, identifying their own behavior that may elicit behavior alteration, sharing caring work with others without feeling guilty.

Given these results we may conclude that there is evidence of the importance of the work developed with caregivers and/or relatives to provide advice and support. There may be limitations for the results due to the reduced number of patients; however group numbers in studies in the literature are also low, due to the difficulty of the rehabilitation process. An obstacle is the major difference among the study designs of intervention in degenerative disease patients. A limitation for this study was the absence of a group control that would allow us to compare the combined treatment effect (neuropsychological rehabilitation + cholinesterase inhibitor) with the effect of medication alone. It is important to point out that the measure of success of a rehabilitation strategy is evaluated in daily life rather than in the therapeutic environment, since the aim of this work is to make patients more functional and adapted to their environment.

## Figures and Tables

**Figure 1:**
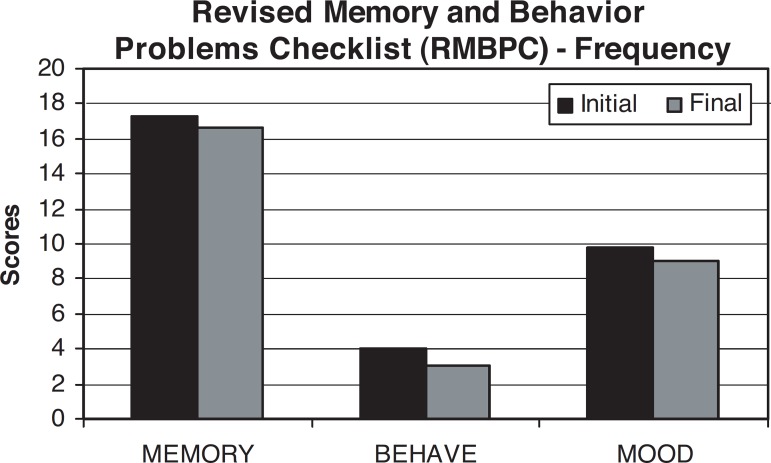
Shows RMBPC frequency scores between initial and second evaluation. We were unable to detect statistical difference between evaluations on observed frequencies: memory (Z=0.314, p=0.753), behaviol (Z= –0.716, p=0.474), mood (Z= –0.401, p=0.688). Frequency of symptoms on RMBPC.

**Figure 2:**
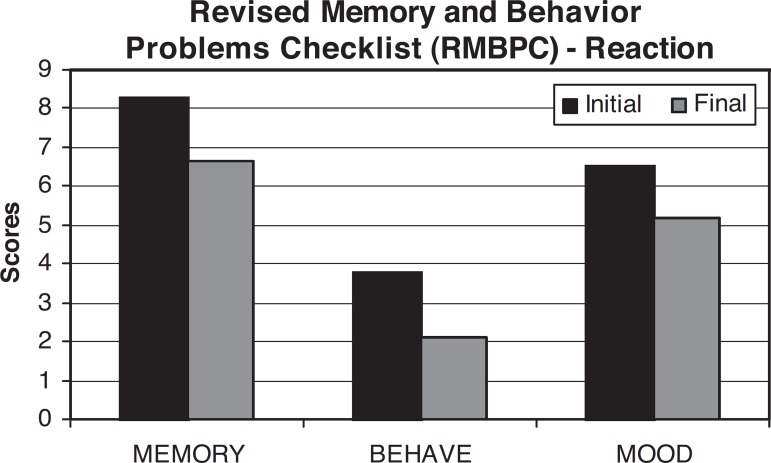
Shows scores for caregiver reaction in relation to patient’s memory, behavior, and mood symptoms on the RMBPC. We noted a significant difference in memory, behavioral and mood scores between the first and second caregiver evaluation (Z= –1. 299, p=0.194), (Z= –1.309, p=0.191) and (Z=0.49, p=0.624). Scores for caregiver reactions in relation to patient’s memory, behavior, and mood symptoms.
